# Synthesis and Characterization of Multilayer 3D Chiral Polymers with Enhanced Optical Properties

**DOI:** 10.3390/molecules30071567

**Published:** 2025-03-31

**Authors:** Sai Zhang, Xiaobei Jin, Daixiang Chen, Qingzheng Xu, Tao Wang, Xiuyuan Qin, Jialing Mao, Yue Zhang, Shenghu Yan, Guigen Li

**Affiliations:** 1 School of Pharmacy, Changzhou University, Changzhou 213164, China; 2 Changzhou Tronly Advanced Electronic Materials Co., Ltd., Changzhou 213011, China; 3 School of Chemistry and Chemical Engineering, Nanjing University, Nanjing 210093, China; 4 School of Life and Science, Nanjing Normal University, Nanjing 210046, China; 5 School of Environmental Science and Engineering, Changzhou University, Changzhou 213164, China; 6 Department of Chemistry and Biochemistry, Texas Tech University, Lubbock, TX 79409-1061, USA

**Keywords:** multilayer 3D chiral polymer, aggregation-induced emission

## Abstract

This study reports the synthesis of novel multilayer 3D chiral polymers using 2,2′-(2,7-Naphthalenediyl)bis[4,4,5,5-tetramethyl-1,3,2-dioxaborolane] and 1,8-dibronaphthalene along with its derivatives as key precursors. Comprehensive characterization was performed using nuclear magnetic resonance (NMR), gel permeation chromatography (GPC), photoluminescence, ultraviolet (UV) spectroscopy, scanning electron microscopy (SEM), polarimetry, dynamic light scattering (DLS), and thermogravimetric analysis (TGA). Notably, the polymers exhibited remarkable aggregation-induced emission (AIE) and aggregation-induced polarization (AIP) phenomena, revealing enhanced luminescence and optical activity in aggregated states. These findings underscore the potential of these chiral polymers for applications in optoelectronics and advanced sensing technologies, highlighting the intricate relationship between molecular structure and optical behavior.

## 1. Introduction

Chiral polymers have emerged as a significant area of research due to their unique optical properties and potential applications across various domains, including optoelectronics [1,2,3,4], photonics [5,6,7,8], and asymmetric synthesis [9,10,11,12]. The chirality of these materials allows them to interact with polarized light in distinctive ways, making them valuable for applications such as circularly polarized light emitters and sensors. Among the various chiral structures, multilayer 3D chiral polymers [13,14,15,16] represent a novel class that not only enhances the inherent chiral characteristics but also introduces complex interactions that can be finely tuned through structural design.

This synthesis of multilayer 3D chiral polymers typically involves the careful selection of starting materials that possess intrinsic chirality and facilitate the formation of robust architectures. In this study, we report the synthesis of several new multilayer 3D chiral polymers using 2,2′-(2,7-Naphthalenediyl)bis[4,4,5,5-tetramethyl-1,3,2-dioxaborolane] and 1,8-dibronaphthalene along with its derivatives as key building blocks. The choice of these materials is driven by their favorable electronic properties and structural motifs that can promote effective chiral interactions. The incorporation of naphthalene units is particularly advantageous due to their planar structure and ability to engage in π–π stacking, which is crucial for the formation of ordered multilayer architectures.

In recent years, the phenomenon of aggregation has garnered significant interest in the field of polymer science [17,18,19,20,21,22,23,24,25,26,27]. Aggregation in this context refers to the observation that certain fluorophores exhibit enhanced luminescence upon aggregation, which contrasts with the traditional aggregation-caused quenching (ACQ) effect typically seen in many organic compounds [28,29]. This unique property is particularly advantageous for the development of luminescent materials that can be utilized in various applications, including light-emitting diodes and bioimaging [30,31,32]. The phenomenon of aggregation-induced polarization [13,14,15,16,33] has also been observed in chiral systems, wherein the optical activity of the polymer is significantly enhanced in the aggregated state. AIP in multilayer chiral polymers presents an exciting opportunity to explore novel optical behaviors that can be harnessed for advanced applications.

To characterize the synthesized polymers, we employed a variety of analytical techniques, including nuclear magnetic resonance (NMR) spectroscopy, gel permeation chromatography (GPC), photoluminescence measurements, ultraviolet (UV) spectroscopy, scanning electron microscopy (SEM), dynamic light scattering (DLS), thermogravimetric analysis (TGA), and polarimetry. NMR spectroscopy elucidates the molecular structures and confirms the incorporation of chiral building blocks. GPC provides crucial data on molecular weight distribution and polydispersity, essential for correlating polymer structure with physical properties. Photoluminescence and UV spectroscopy reveal enhanced luminescence in aggregated states, highlighting their potential as efficient luminescent materials. Additionally, SEM and DLS provide insights into morphology, while TGA assesses thermal stability. Cyclic voltammetry (CV) elucidates redox behavior and electrochemical stability, and polarimetric measurements quantify the optical activity, revealing insights into chirality.

**Figure 1:**
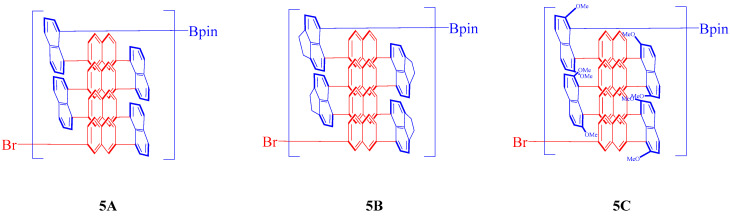
Synthetic multilayer 3D chiral investigated polymers.

This research presents a comprehensive investigation of novel multilayer 3D chiral polymers synthesized from carefully selected starting materials (Figure 1). By employing rigorous characterization techniques, we aim to elucidate the intriguing phenomena of aggregation and aggregation-induced polarization observed in these materials, thereby deepening our understanding of chiral polymers and their potential applications. The findings of this study not only enhance our fundamental knowledge of chiral polymers but also highlight the critical role of structural design in optimizing optical properties for advanced material applications.

## 2. Experimental Section

### 2.1. Materials and Instrumentation

All procedures were conducted under an inert argon atmosphere, utilizing anhydrous solvents in oven-dried glassware, with magnetic stirring throughout. Solvents, liquids, and solutions were introduced via syringes, stainless steel or polyethylene cannulas, and rubber septa, or through a gentle argon counter-flow. Cooling baths of ice/water (0 °C) or dry ice/acetone (−78 °C) were prepared in Dewar containers, while heated oil baths were employed for high-temperature reactions. Solvent removal was achieved using rotary evaporators at temperatures between 40 and 65 °C. The yields reported are chromatographic and NMR yields, determined separately.

All commercially available reagents were utilized as received without further purification. Solvents, including methanol (CH_3_OH), toluene, ethyl acetate (EA), diethyl ether, dichloromethane (DCM), dioxane, and acetone, were used without additional purification. Tetrahydrofuran (THF) and DCM were supplied through an Innovation Technology solvent delivery system. Proton nuclear magnetic resonance (¹H NMR) spectra were recorded on a 400 MHz spectrometer, using tetramethylsilane (TMS) as an internal standard. The chemical shifts were referenced to the residual solvent signal (7.26 ppm for CDCl_3_), with the solvent signal also used for ^13^C NMR spectra (7.16 ppm for CDCl_3_). Chemical shifts were reported in parts per million (ppm), with data described in terms of chemical shift, multiplicity (singlet, doublet, triplet, and multiplet), coupling constants (J, Hz), and integration. Gel permeation chromatography (GPC) data were collected using a TOSOH EcoSEC HLC-8420 GPC (Tokyo, Japan), equipped with a dual-flow refractive index detector and a UV detector for UV–visible polymers. The installed columns cover a molecular weight range of 500–107 kDa, with samples collected for 25 min at a flow rate of 0.7 mL/min. Calibration was performed using polystyrene (PS) standards. Photoluminescence spectra and UV–visible absorption were obtained using Shanghai Lengguang F98 and Shanghai Lengguang 759s spectrophotometers (Shanghai, China). Particle sizes were measured using a nano laser particle size analyzer (Winner 802, Jinan Winner Particle Instrument Stock Co., Ltd., Jinan, China). The thermogravimetric analysis was conducted with a simultaneous thermal analyzer (FBS-STA 200JG, Xiamen Furbs Testing Equipment Co., Ltd., Xiamen, China). Cyclic voltammetry was conducted using the DH7000 electrochemical measurement system (Jiangsu Donghua Analytical Instrument Co., Ltd., Taizhou, China). The selected samples were prepared in degassed dichloromethane (DCM) containing 0.1 M Bu_4_NPF_6_ as the supporting electrolyte at 298 K. A conventional three-electrode cell configuration was employed, consisting of a glassy carbon disc working electrode (with a defined geometric surface area), a platinum wire counter electrode, and an Ag/AgNO_3_ (non-aqueous) reference electrode. The scan rate for the cyclic voltammetry measurements was set at 100 mV/s.

### 2.2. Synthetic Procedure of Polymers ***5A***, ***5B***, and ***5C***

**Figure 2:**
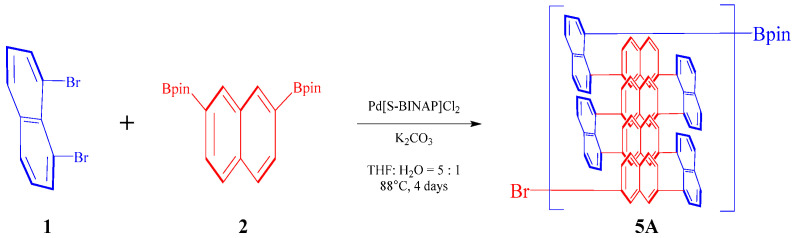
Synthetic method of polymer **5A**.

A 50 mL oven-dried round-bottom flask was charged with 2,2′-(2,7-Naphthalenediyl)bis[4,4,5,5-tetramethyl-1,3,2-dioxaborolane] **2** (316.8 mg, 0.8 mmol, 1 equiv), 1,8-dibromonaphthalene **1** (228.8 mg, 0.8 mmol, 1.0 equiv), Pd[S-BINAP]Cl_2_ (32 mg, 0.04 mmol), and K_2_CO_3_ (439 mg, 3.2 mmol). The flask was then filled with 2 mL of water and 10 mL of tetrahydrofuran (THF) (Figure 2). After vacuum degassing, argon was introduced into the flask. The mixture was heated at 88 °C for over 4 days. Following this period, the mixture was allowed to cool to room temperature. Subsequently, the resultant mixture was added to methanol (MeOH) containing hydrochloric acid (HCl). The precipitated components were filtrated through a Buchner funnel and thoroughly rinsed with methanol and water. The polymer solid **5A** was then dried to yield a dark green product (371 mg, 68% yield).

A 50 mL oven-dried round-bottom flask was charged with 2,2′-(2,7-Naphthalenediyl)bis[4,4,5,5-tetramethyl-1,3,2-dioxaborolane] **2** (316.8 mg, 0.8 mmol, 1 equiv), 5,6-dibromo-1,2-dihydroacenaphthylene **5** (249.6 mg, 0.8 mmol, 1.0 equiv), Pd[S-BINAP]Cl_2_ (32 mg, 0.04 mmol), and K_2_CO_3_ (439 mg, 3.2 mmol). The flask was then filled with 2 mL of water and 10 mL of tetrahydrofuran (THF). After vacuum degassing, argon was introduced into the flask. The mixture was heated at 88 °C for over 96 h. Following this period, the mixture was allowed to cool to room temperature. Subsequently, the resultant mixture was added to methanol (MeOH) containing hydrochloric acid (HCl). The precipitated components were filtrated through a Buchner funnel and thoroughly rinsed with methanol and water. The polymer solid **5C** was then dried to yield a yellow-green product (312 mg, 55% yield).

**Figure 3:**
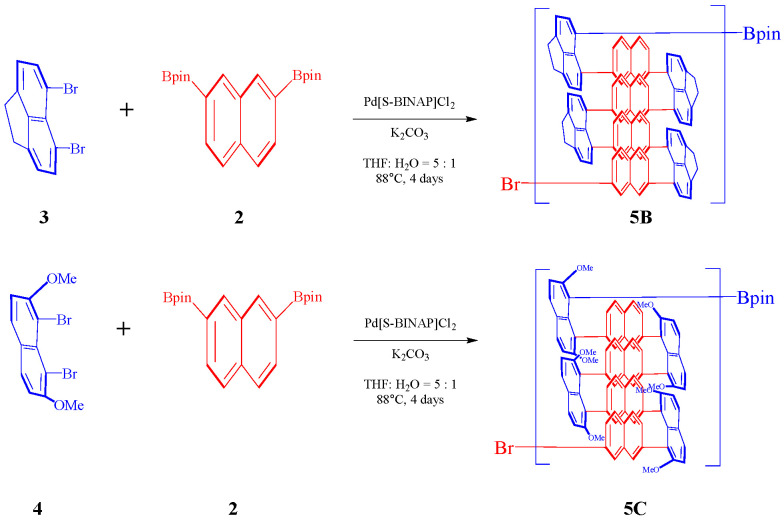
Synthetic method of polymers **5B** and **5C**.

A 50 mL oven-dried round-bottom flask was charged with 2,2′-(2,7-Naphthalenediyl)bis[4,4,5,5-tetramethyl-1,3,2-dioxaborolane] **2** (316.8 mg, 0.8 mmol, 1 equiv), 1,8-dibromo-2,7-dimethoxynaphthalene **4** (276.8 mg, 0.8 mmol, 1.0 equiv), Pd[S-BINAP]Cl_2_ (32 mg, 0.04 mmol), and K_2_CO_3_ (439 mg, 3.2 mmol). The flask was then filled with 2 mL of water and 10 mL of tetrahydrofuran (THF). After vacuum degassing, argon was introduced into the flask. The mixture was heated at 88 °C for over 96 h. Following this period, the mixture was allowed to cool to room temperature. Subsequently, the resultant mixture was added to methanol (MeOH) containing hydrochloric acid (HCl). The precipitated components were filtrated through a Buchner funnel and thoroughly rinsed with methanol and water. The polymer solid **5C** was then dried to yield a yellow-green product (379 mg, 61% yield) (Figure 3).

### 2.3. Schematic Procedure of Purifying Synthetic Multilayer 3D Chiral Polymers

After the completion of the reaction, the mixture was allowed to cool to room temperature. We then performed the following purification steps:

(1) Precipitation: The reaction solution was slowly poured into a mixture of methanol (MeOH) and 6 N hydrochloric acid (HCl) (25 mL MeOH/5 mL HCl). This step facilitated the precipitation of the polymer, resulting in the formation of a yellow solid.
(2) Stirring: The precipitated mixture was stirred for 30 min at room temperature to ensure complete coagulation of the polymer.
(3) Filtration: The solid polymer was recovered by filtration using a Buchner funnel.
(4) Washing: To ensure the removal of any unreacted materials and byproducts, the precipitated polymer was washed with 2 N HCl (10 mL), followed by deionized water (10 mL).
(5) Drying: Finally, the washed polymer was dried in an oven at 56 °C overnight to remove residual moisture and solvents, yielding the final polymer solid.

## 3. Results and Discussion

### 3.1. GPC and Polarization Analysis

The gel permeation chromatography (GPC) analysis of the synthetic multilayer 3D chiral polymers reveals significant insights into their molecular weight characteristics and structural implications (Table 1). For polymer **5A**, which incorporates naphthalene units, the weight-average molecular weight (Mw) is 115,453 g/mol, with a number-average molecular weight (Mn) of 33,934 g/mol and a polydispersity index (PDI) of 3.402 (Supplementary Materials, Figure S4). The relatively high PDI indicates a broad molecular weight distribution, which is typical for polymers synthesized via step-growth polymerization. The theoretical layer count of 89 suggests a well-defined multilayer structure, which is crucial for its chiral properties. In polymer **5B**, featuring naphthalene-dihydroacenaphthylene units, the Mw is 116,561 g/mol and the Mn is 33,209 g/mol, yielding a PDI of 3.510 (Figure S4). The introduction of dihydroacenaphthylene likely enhances the polymer’s rigidity and contributes to its chiral architecture, as the fused aromatic system can promote π–π stacking interactions, potentially influencing the multilayer formation. The theoretical layer count of 76 indicates a slight reduction in layering compared to polymer **5A**, which may correlate with steric effects introduced by the dihydroacenaphthylene moiety. Polymer **5C**, containing methoxy-substituted naphthalene, exhibits an Mw of 116,894 g/mol and an Mn of 33,272 g/mol, with a PDI of 3.512 (Figure S3). The methoxy group can enhance solubility and influence the polymer’s electronic properties, potentially facilitating better packing and layer formation. The similar PDI values across the three polymers suggest consistent synthetic conditions, while variations in Mw and layer counts reflect the influence of specific substructures on the overall polymer architecture.

Polymer **5A**, which consists solely of naphthalene units, exhibits the highest number-average molecular weight (Mn), the largest theoretical layer count, and the lowest polydispersity index (PDI) among the three polymers. The elevated Mn of **5A** (33,934 g/mol) can be attributed to the simplicity and uniformity of its naphthalene-based structure, which likely facilitates a more efficient and consistent polymerization process. This uniformity minimizes chain termination and branching, resulting in longer, more homogeneous polymer chains. The theoretical layer count of 89 for polymer **5A** indicates a well-defined multilayer architecture, which is a direct consequence of the regularity in its naphthalene structure. The absence of bulky substituents allows for optimal stacking and alignment of the polymer chains, enhancing the formation of distinct layers. Additionally, the lower PDI of 3.402 suggests a narrower molecular weight distribution, indicative of a more controlled polymerization process. In contrast, polymers **5B** and **5C**, which incorporate bulky dihydroacenaphthylene and methoxy groups, respectively, experience steric hindrance and potential chain irregularities, leading to increased molecular weight distributions and lower theoretical layer counts. These factors collectively elucidate the superior characteristics of polymer **5A** in terms of molecular weight, layer formation, and uniformity.

The synthesized polymers are primarily composed of naphthalene, bromine (Br), methoxy, and boric esters, reflecting a carefully designed elemental composition that enhances their chiral properties. This fundamental composition plays a crucial role in the polymers’ structural integrity and contributes to their unique optical characteristics. Following the analysis of their elemental makeup, we observed significant optical rotation in these polymers, indicative of their chiral nature and potential applications. The ability to finely tune the elemental composition allows for the optimization of their optical activities, which is essential for advancing their use in optoelectronic devices and other chiral applications.

The optical rotation values of the synthesized multilayer 3D chiral polymers demonstrate a clear correlation between structural composition and chiroptical properties. Polymer **5A**, which consists solely of naphthalene, exhibits the highest optical rotation of +24. The notable optical activity of these polymers is primarily due to the inherent chirality and efficient π–π stacking of naphthalene units, which support a defined chiral arrangement for strong optical rotation. Polymer **5B**, incorporating naphthalene-dihydroacenaphthylene, exhibits a reduced optical rotation of +11, as the bulky side group introduces steric hindrance and disrupts chiral uniformity, resulting in weaker π–π interactions. Polymer **5C**, with a methoxy group, shows the lowest optical rotation of +3. The methoxy group adds flexibility and steric bulk, further compromising the planar structure and reducing chiral interactions, highlighting the importance of structural integrity in chiroptical properties.

### 3.2. Characteristics of UV-Vis and Photoluminescence

The UV-Vis absorption spectra of multilayer 3D chiral polymers, particularly those with naphthalene derivatives, reveal valuable information about their electronic properties and aggregation behavior. Polymers **5A** and **5B** show strong absorbance from 270 to 370 nm, indicating π–π* transitions related to the naphthalene chromophore. Polymer **5A** displays a distinct feature between 300 and 325 nm, suggesting aggregation. The tailing in both polymers indicates significant intermolecular interactions, while polymer **5C**, with a methoxy group, shows an additional peak from 375 to 450 nm due to enhanced intramolecular charge transfer.

The ultraviolet (UV) spectral analysis of multilayer 3D chiral polymers shows distinct electronic transitions linked to their molecular structure. Polymer **5C**, containing dimethoxynaphthalene, displays a clear π–π* transition, indicating strong electronic interactions, enhanced conjugation, and electron delocalization from methoxy substituents that stabilize excited states.

**Figure 4:**
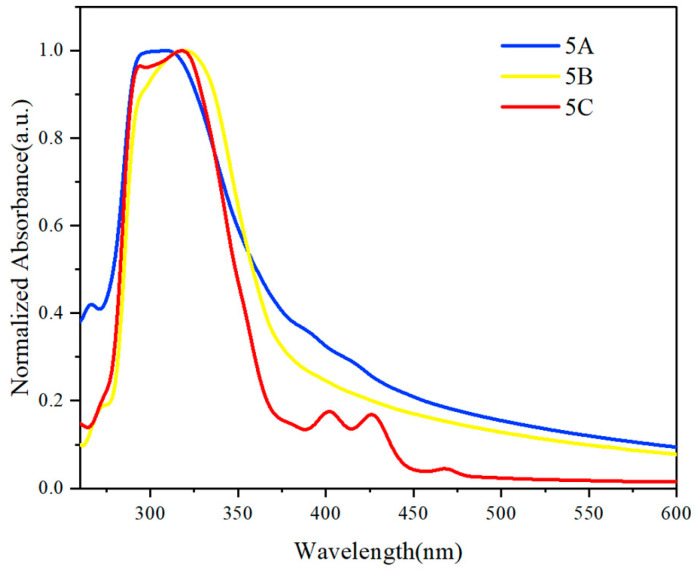
UV-Vis absorption spectrum of **5A**, **5B**, and **5C**. Concentration: 0.05 mg/mL in chloroform.

Polymers **5A** and **5B**, both of which are based on naphthalene and its derivatives, display relatively similar UV spectra, indicating analogous electronic environments (Figure 4). The lack of significant differentiation between these two polymers implies that the structural modifications introduced in polymer **5B**, through the incorporation of dihydroacenaphthylene, do not substantially alter the electronic transitions compared to the naphthalene framework of polymer **5A**. This observation underscores the role of specific functional groups in modulating the optical properties of multilayer chiral polymers.

The excitation wavelengths of the multilayer 3D chiral polymers exhibit distinct variations that reflect their unique molecular compositions and structural characteristics. Polymer **5A**, which is naphthalene-based, demonstrates an excitation wavelength of 330 nm, indicative of its relatively higher energy electronic transitions. In contrast, both polymer **5B**, incorporating naphthalene and dihydroacenaphthylene, and polymer **5C**, composed of naphthalene and dimethoxynaphthalene, exhibit excitation wavelengths of 426 nm. This shift to a longer wavelength suggests a lower energy transition, likely due to the influence of the additional substituent groups in polymers **5B** and **5C**, which can alter the electronic distribution and conjugation length. The similarity in the excitation wavelengths of polymers **5B** and **5C** highlights the role of structural modifications in tuning the electronic properties of multilayer chiral polymers.

**Figure 5:**
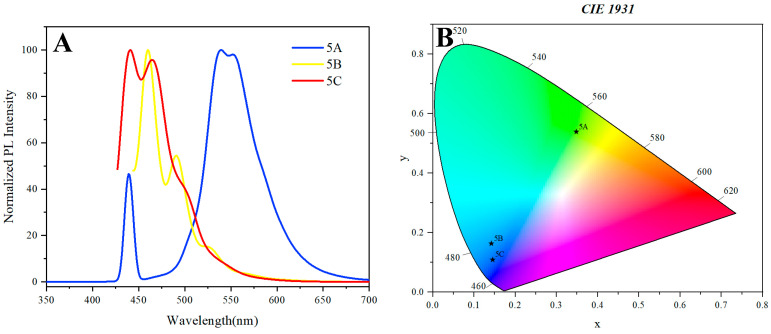
(**A**) Photoluminescence spectrum of **5A**, **5B**, and **5C**. Concentration: 0.05 mg/mL in THF. (**B**) CIE 1931 coordinates of polymer **5A**, **5B**, and **5C**. (Excited wavelengths: 330 nm (**5A**) and 426 nm (**5B** and **5C**)).

In polymer **5A**, the presence of two prominent peaks at approximately 440 nm and 540 nm is noteworthy, with the latter exhibiting significantly greater intensity, suggesting a specific electronic transition (Figure 5A). The peak at 540 nm is indicative of a more efficient radiative transition, potentially involving an intramolecular charge transfer (ICT) state or an excited state stabilized by the polymer’s conformation. The pronounced intensity of this peak reflects a favorable environment for emission, attributed to reduced non-radiative decay pathways in the excited state. The peak at 440 nm is likely associated with Raman scattering rather than a direct electronic transition related to the naphthalene core. This distinction is crucial, as it raises questions about the interpretation of the nature of the other bands in the luminescence spectra of the investigated polymers.

For polymer **5B**, which incorporates both naphthalene and acenaphthene, the photoluminescence (PL) spectrum reveals peaks at 461 nm and 492 nm, with the peak at 461 nm being more intense (Figure 5A). The observed shift in peak position compared to polymer **5A** is attributed to the introduction of acenaphthene, which modifies the electronic environment and introduces new pathways for exciton relaxation. The differences in peak intensities suggest that the acenaphthene moiety enhances non-radiative decay processes or competes with the radiative transition-associated naphthalene unit, resulting in a relative suppression of emission at longer wavelengths.

Polymer **5C**, featuring a methoxy substituent, displays peaks at 441 nm and 464 nm, with the peak at 441 nm being slightly more intense. The methoxy group, as an electron-donating substituent, significantly influences the electronic properties of the polymer. The proximity of these two peaks indicates a closer energy level alignment, which facilitates rapid exciton migration between states. The relatively balanced intensities of the peaks suggest that the electronic transitions in this polymer are more evenly matched, potentially due to the stabilization of excited states through intramolecular interactions.

### 3.3. Analysis of Aggregation Phenomenon

Aggregation refers to the process where individual molecules come together to form larger assemblies. In the context of fluorescent materials, this phenomenon can lead to unique optical properties. Specifically, certain fluorescent molecules exhibit increased light emission when aggregated, a behavior that contrasts with conventional fluorescent compounds, which often experience quenching in similar conditions. Aggregated structures are gaining significance in various applications, including bioimaging, sensor technologies, and organic light-emitting diodes (OLEDs). Additionally, the aggregation of molecules can enhance molecular polarization, which in turn improves the non-linear optical properties of materials used in photonic devices.

**Figure 6:**
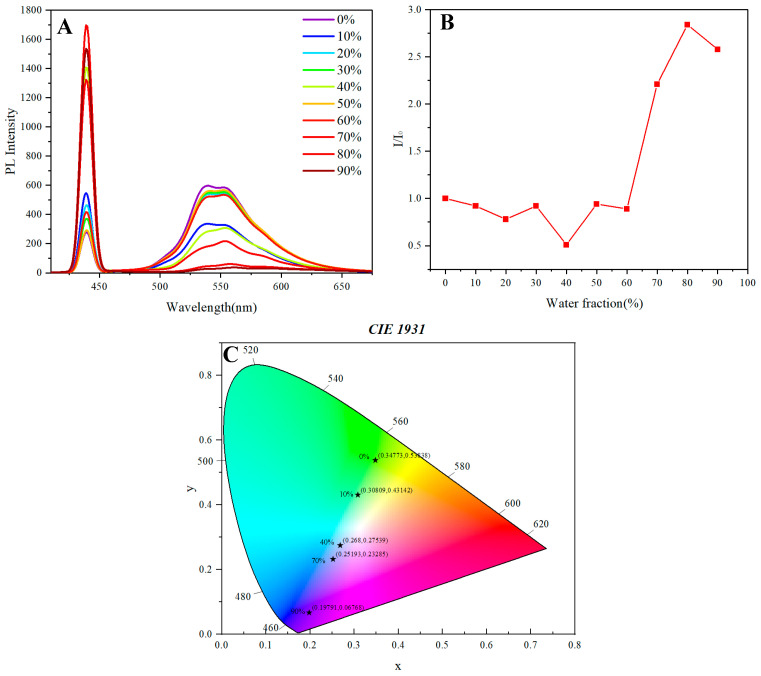
(**A**) PL spectra of **5A** in THF/water mixtures with different water fractions (f_w_); c = 0.05 mg mL^−1^; inset: fluorescence photographs of **5A** in a THF/water system. (**B**) Stern–Volmer plots of I/I_0_ vs. DI water fraction of **5A**. (**C**) CIE 1931 coordinate of 5A in different DI water fractions. (Excited wavelength: 330 nm (**5A**)).

The fluorescence properties of synthetic multilayer 3D chiral polymers, especially those containing naphthalene-based polymer **5A**, display interesting behaviors under different water fractions. Notably, a strong aggregation effect is observed at water fractions between 60% and 90%, as indicated by a significant enhancement of the emission peak at 440 nm (Figure 6A).

In contrast, the fluorescence peak of polymer **5A** observed at approximately 550 nm demonstrates an aggregation-caused quenching (ACQ) phenomenon (Figure 6A). This quenching behavior, however, does not follow a straightforward stepwise progression. Instead, it appears to mirror the behavior of the first peak in the water fraction range of 0% to 50%. This observation suggests that the polymer’s fluorescence characteristics are highly sensitive to the aggregation state of the chromophores, which is influenced by the solvent environment.

The Stern–Volmer plot serves as a valuable tool for elucidating the relationship between the degree of aggregation and fluorescence quenching (Figure 6B). The plot effectively reflects the total tendency of aggregation and quenching phenomena, providing insights into the dynamic interactions between the polymer chains and the solvent molecules. As the water fraction increases, the polymer chains likely undergo conformational changes that promote aggregation, leading to a decrease in the effective fluorescence quantum yield. The non-linear behavior observed in the Stern–Volmer plot indicates complex interactions that may involve both static and dynamic quenching mechanisms.

The chromatic shifts observed during the fluorescence measurements further highlight the sensitivity of the polymer’s optical properties to changes in the solvent composition. The coordinates of the emission spectra at varying water fractions—(0.34773, 0.53838), (0.30809, 0.43142), (0.268, 0.27539), (0.25193, 0.23285), and (0.19791, 0.06768) corresponding to water fractions of 0%, 10%, 40%, 70%, and 90%, respectively—demonstrate a clear trend of color transfer. This chromatic transition may be attributed to the alterations in the electronic environment surrounding the chromophores as they aggregate in response to increasing water content.

The initial water fraction (0%) corresponds to a relatively hydrophobic environment, which likely facilitates the solubilization of the naphthalene units, thereby enhancing their emission properties. As water is gradually introduced, the increasing polarity of the solvent could disrupt the π–π stacking interactions between the naphthalene moieties. This disruption is associated with a noticeable red shift in the emission spectra, which reflects the alterations in the electronic environment of the chromophores.

The analysis of the chromaticity coordinates reveals a systematic decrease in both the x and y values, suggesting a transition from vibrant emission to more subdued fluorescence as aggregation occurs and quenching mechanisms become prominent. Particularly at higher water fractions (70% and 90%), the significant drop in chromaticity coordinates indicates that the polymer predominantly adopts an aggregated state. In this context, the effective conjugation length of the chromophores is reduced due to enhanced intermolecular interactions, leading to a substantial decrease in fluorescence intensity and a notable shift in the emission profile.

It is critical to emphasize that the initial observations of sharp peaks could be influenced by Raman scattering. As such, the evaluation of the AIE effect should focus on distinct luminescence bands. Our subsequent analysis of the luminescence in the blue-green region of the spectrum corroborates the notion that a true AIE effect is not manifested, as the intensity decreases sharply with increasing water content.

The interplay between aggregation-induced emission (AIE) and aggregation-caused quenching (ACQ) in the naphthalene-containing polymer **5A** underscores the importance of molecular design in tuning the optical properties of chiral polymers. The observed fluorescence behavior is not merely a result of aggregation; it also reflects the intricate molecular interactions and conformational changes that occur in response to solvent polarity.

**Figure 7:**
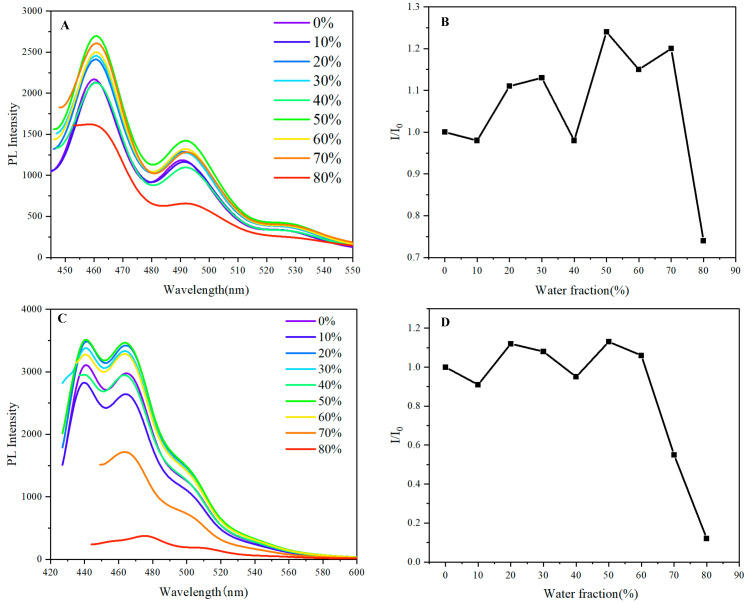
(**A**,**C**) PL spectra of **5B** and **5C** in THF/water mixtures with different water fractions (f_w_); c = 0.05 mg mL^−1^; inset: fluorescence photographs of **5B** and **5C** in the THF/water system. (**B**,**D**) Stern–Volmer plots of I/I_0_ vs. DI water fraction of **5B** and **5C**. (Excitation wavelength: 426 nm (**5B** and **5C**)).

In our subsequent study, we further explore the impact of aggregation on the optical properties of naphthalene-containing multilayered 3D chiral polymers. Our results reveal that different polymer structures exhibit varying degrees of aggregation-related emission behaviors. Specifically, polymer **5B**, which contains naphthalene-dihydroacenaphthylene units, demonstrates a significant response in luminescence as the water fraction varied from 0% to 70%. Notably, there is a marked increase in luminescence upon aggregation; however, we also observe a decrease in fluorescence intensity at a water fraction of 40% (Figure 7). This nuanced observation suggests that the interactions within the polymer structure can be influenced by the solvent environment, leading to complex changes in the emission characteristics as aggregation occurs.

The pronounced aggregation response of polymer **5B**, characterized by enhanced luminescence as the water fraction increases, indicates that its multilayered architecture supports effective π–π stacking and spatial confinement. This facilitates significant luminescence enhancement upon aggregation, signaling a favorable interaction among the naphthalene units. However, the decrease in fluorescence intensity observed at a water fraction of 40% suggests a critical threshold: as hydrophilicity increases, it disrupts optimal molecular packing, resulting in diminished emission efficiency.

In contrast, polymer **5C**, which features naphthalene-methoxy units, exhibits a subtler aggregation effect. The presence of the methoxy substituent introduces steric hindrance that limits effective π–π interactions and, consequently, the degree of luminescence enhancement when aggregates form. The methoxy group influences the polymer’s conformational adaptability and packing efficiency, leading to a less pronounced aggregation effect compared to polymer **5B**.

While it is important to delineate between the mechanisms of luminescence and Raman scattering, our analyses explicitly focus on the luminescence properties observed in the blue-green region of the spectrum. The sharp decrease in intensity with increasing water content for both polymers underscores the complexities within the aggregation phenomena and suggests that an AIE effect could not be present in this system. While polymer 5B shows initial enhancement in luminosity upon aggregation, the observed reduction at 40% water fraction warrants careful consideration regarding the specific conditions under which AIE could be expected.

Our findings highlight the intricate interplay between molecular design, steric effects, and solvent environment in determining the aggregation behavior and optical properties of multilayered 3D chiral polymers. These insights provide a fundamental basis for advancing the development of materials with tailored emission characteristics for applications in optoelectronics and sensing.

Aggregation-induced polarization is a distinctive optical phenomenon observed in certain luminescent materials, characterized by enhanced emission intensity upon aggregation. Unlike conventional fluorescence, which often diminishes with increasing concentration due to self-quenching, AIP materials exhibit a marked increase in luminescence, attributed to the suppression of non-radiative decay pathways and the stabilization of excited states. This intriguing behavior is rooted in the intricate interactions among chromophores, such as π–π stacking. The unique properties of AIP have significant implications for the development of advanced optical devices, sensors, and biomedical applications, offering opportunities for improved sensitivity and specificity in various fields.

**Figure 8:**
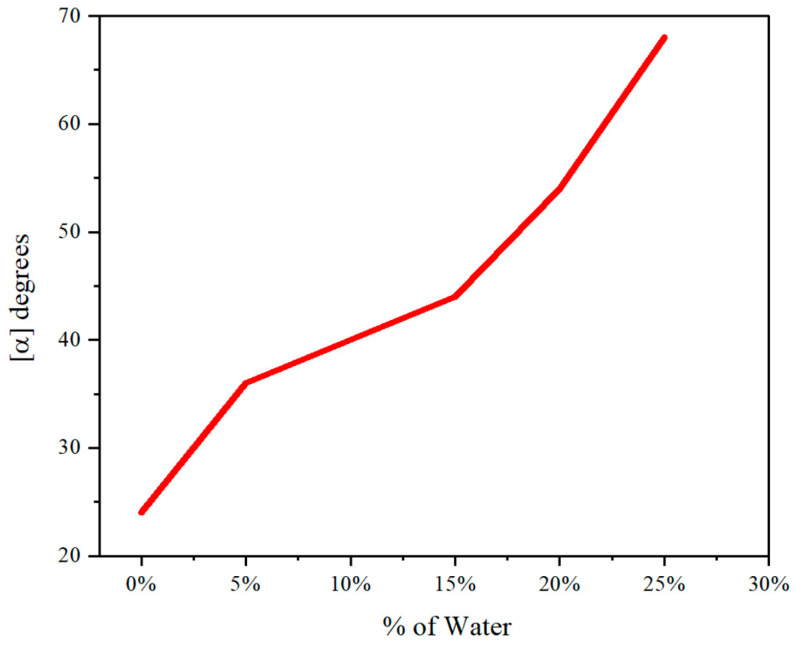
Aggregation-induced polarization of chiral folding polymer **5A** in THF; c = 0.5 mg/mL.

In the investigation of aggregation-induced polarization within multilayer 3D polymer **5A**, a notable increase in optical rotation was observed, escalating from +24 to +66 as the water fraction was incrementally raised from 0% to 25% (Figure 8). This pronounced enhancement in optical activity can be attributed to the aggregation of chromophores within the polymer matrix, which facilitates stronger intermolecular interactions and promotes the stabilization of excited states, thereby amplifying the polarization effect. The increase in water fraction likely induces a favorable microenvironment that enhances the aggregation process, leading to the observed optical rotation enhancement. However, a critical instability in the optical rotation was noted at a water fraction of 30%, suggesting a threshold beyond which the system undergoes significant structural or conformational changes. At this concentration, the polymer chains may experience increased solvation and mobility, disrupting the optimal aggregation state necessary for sustained AIP. The introduction of excess water could lead to competitive interactions, such as solvation effects or phase separation, which may detrimentally affect the chromophore alignment and aggregation. This phenomenon underscores the delicate balance between solvent interactions and aggregation dynamics, highlighting the need for precise control over environmental conditions to optimize AIP in polymeric systems.

### 3.4. Analysis of Particle Size

**Figure 9:**
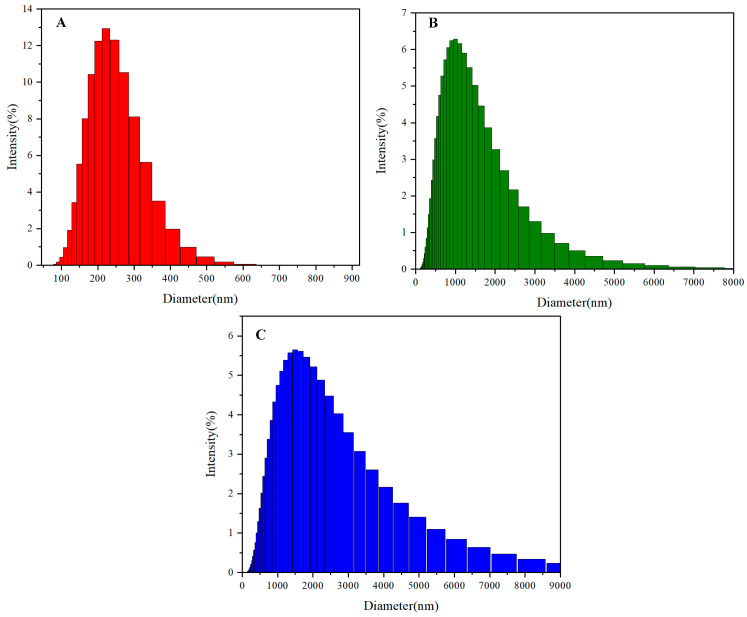
(**A**–**C**) Dynamic light scattering analysis of **5A**, **5B**, and **5C** in THF.

The dynamic light scattering (DLS) measurements of the multilayer 3D chiral polymers synthesized from 2,2′-(2,7-Naphthalenediyl)bis[4,4,5,5-tetramethyl-1,3,2-dioxaborolane] **2** and 1,8-dibronaphthalene derivatives **1**, **3**, and **4** reveal intriguing insights into the influence of molecular architecture and substructure on particle size distribution. The observed particle size of 222 nm for the benzene and naphthalene-containing multilayer 3D chiral polymer indicates a relatively compact structure, likely attributed to the efficient packing and π–π stacking interactions inherent to these aromatic components. The presence of naphthalene, with its larger conjugated system compared to benzene, facilitates stronger intermolecular interactions, contributing to the stabilization of smaller aggregates. In contrast, polymers containing benzene, naphthalene, and hydroacene exhibit a significant increase in particle size, with the highest intensity percentage being around 1000 nm (Figure 9). This increase can be attributed to the additional structural complexity introduced by hydroacene, which promotes the formation of larger aggregates due to enhanced hydrophobic interactions and the potential for extended π-conjugation. The bulky hydroacene moieties may disrupt the compact packing observed in simpler structures, leading to the formation of larger, less densely packed aggregates.

Furthermore, the polymer incorporating benzene, naphthalene, and methoxy groups shows an even larger particle size of approximately 1500 nm. The methoxy substituent, being an electron-donating group, can alter the electronic properties and solubility of the polymer, potentially leading to increased steric hindrance and reduced intermolecular interactions. This effect can promote the formation of larger aggregates as a result of weaker cohesive forces within the polymer matrix.

**Figure 10:**
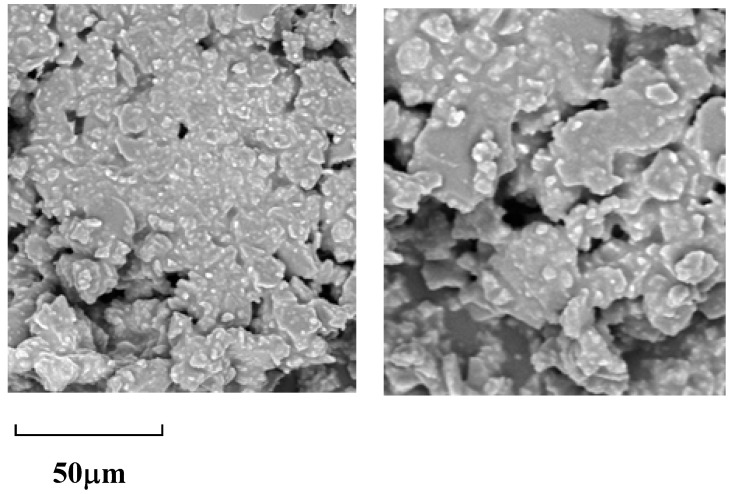
SEM picture of **5A** (**left**) and **5B** (**right**).

In the analysis of the scanning electron microscopy (SEM) images of the multilayer 3D chiral polymer, distinct small particles were observed within each layer, exhibiting a propensity to aggregate (Figure 10). This phenomenon corroborates the hypothesized multilayer structure of the polymer. The presence of these discrete particles suggests a hierarchical organization, where individual layers are not only formed but also interact through aggregation, potentially enhancing the overall structural integrity and functionality of the material. The layered arrangement may facilitate specific interactions among the particles, contributing to the chiral properties of the polymer. Furthermore, the observed morphology provides insights into the processing conditions and the self-assembly mechanisms at play during the formation of the multilayer architecture. This layered aggregation is critical for understanding the material’s properties and behaviors, paving the way for future applications in areas such as photonics and catalysis, where chiral structures can play a pivotal role.

### 3.5. Circular Dichrosim

Circular dichroism (CD) spectroscopy is a pivotal technique used for investigating chiral polymers through the measurement of their differential absorption of left- and right-handed circularly polarized light. The resulting CD spectra unveil crucial insights into the conformational characteristics and secondary structures of the polymers, including aspects such as helicity and aggregation behavior. By analyzing CD signals, researchers can evaluate enantiomeric purity and monitor conformational changes in response to various stimuli, thus enhancing the understanding of the intricate relationship between molecular structure and optical activity in chiral materials.

In this study, we analyzed the optical activity of chiral polymer **5A** in tetrahydrofuran (THF) using CD spectroscopy. A sample solution was prepared at an optimal concentration of 0.5 mg/mL to ensure sufficient signal intensity while minimizing solvent interference. The sample was housed in a quartz cuvette, allowing for the transmission of light across the relevant wavelengths. A monochromatic light source directed circularly polarized light through the sample, and the CD spectrometer measured the differential absorption of left- and right-handed circularly polarized light at each wavelength.

**Figure 11:**
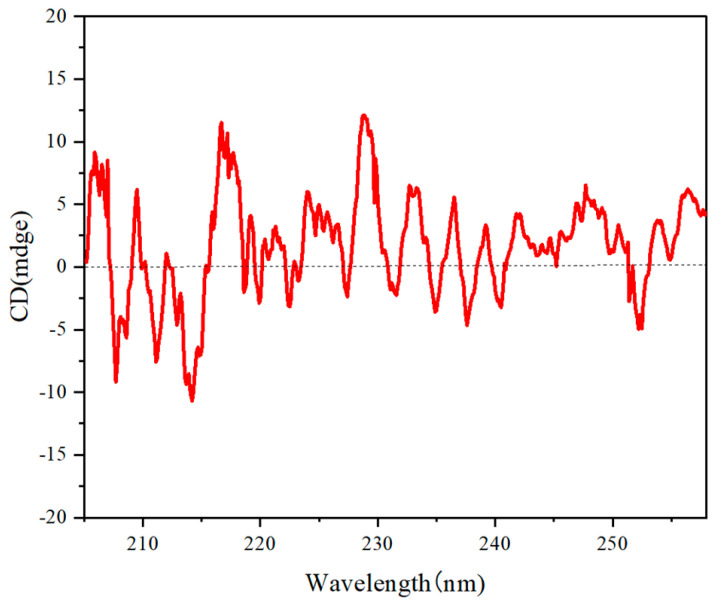
CD spectra of polymer **5A** in THF; c = 0.2 mg/mL.

The CD spectrum of polymer **5A** revealed a prominent optical absorption feature between 210 and 260 nm, which is characteristic of π−π* transitions typically observed in aromatic compounds. Notably, pronounced negative Cotton effects were identified within the wavelength ranges of 206–207 nm, 213–215 nm, 222–225 nm, and 247–252 nm. In contrast, positive Cotton effects were observed in the ranges of 215–218 nm, 228–230 nm, 235–237 nm, and 242–244 nm. These distinctive Cotton effects, illustrated in Figure 11, are indicative of the presence of chirality and provide insights into the molecular conformation and interactions of polymer **5A**.

It is acknowledged that the strong absorption of THF could complicate the analysis of CD signals in the 205–250 nm range. To mitigate this, we carefully selected our measurement conditions and ensured that the concentrations employed did not overwhelm the CD signal with solvent absorption effects. The CD spectrum is thus interpreted within the context of the known refractive and absorption properties of THF, reinforcing the conclusion that the observed Cotton effects are genuine indicators of chirality in polymer **5A**.

These findings not only support the existence of optical activity in this polymer but also contribute to a deeper understanding of its molecular structure and interactions. The distinct Cotton effects observed in the CD spectra of polymer **5A** affirm its chiroptical properties, thereby providing a reliable basis for future studies on the enantiomeric differentiation of chiral materials.

### 3.6. Thermal Properties of Polymers

**Figure 12:**
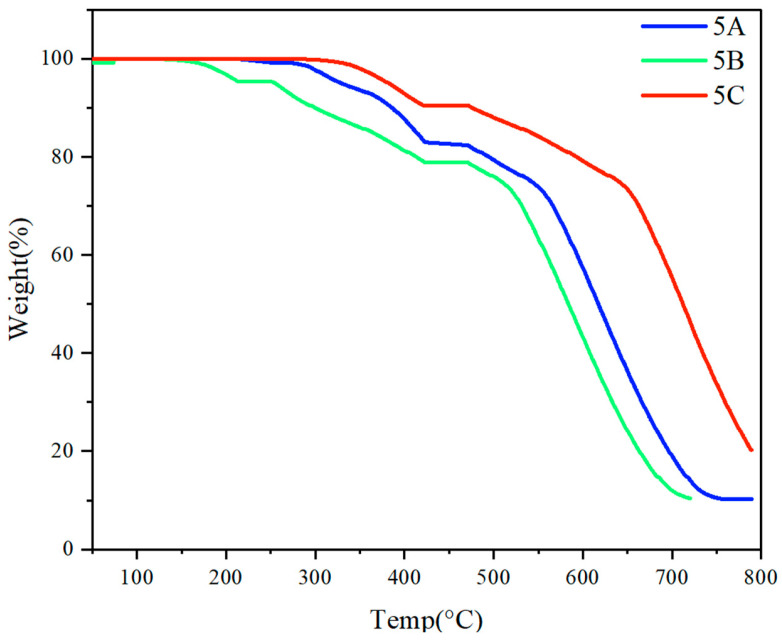
TGA curves of **5A**, **5B**, and **5C**.

The thermogravimetric analysis (TGA) of multilayer 3D chiral polymers reveals significant insights into their thermal stability and potential applications (Figure 12). The benzene and naphthalene-containing polymer exhibits a weight loss of approximately 20% at 400 °C, indicating a relatively lower thermal stability compared to the hydroacene and methoxy-containing polymers, which show only about 15% weight loss under the same conditions. This difference is attributed to the structural characteristics and intermolecular interactions of the respective polymers, with the benzene and naphthalene moieties being more susceptible to thermal degradation. The consistent solid residue of around 10% at 800 °C across all polymer variants suggests a stable inorganic or carbonaceous framework remaining post-degradation. This residual material may be beneficial for applications requiring thermal resistance, such as in coatings or composite materials where high-temperature stability is critical. The reduced weight loss in hydroacene and methoxy-containing polymers indicates enhanced thermal stability, making these materials potentially suitable for applications in electronics or optoelectronics, where prolonged thermal exposure is common. These findings underscore the importance of molecular design in tailoring the thermal properties of chiral polymers for specific industrial applications.

### 3.7. Electrochemical Performance

Cyclic voltammetry (CV) is a highly effective electrochemical technique that plays a pivotal role in the characterization of multilayered three-dimensional (3D) polymers. The significance of CV stems from its capacity to elucidate the redox behavior, charge transport mechanisms, and electrochemical stability of these advanced materials. The simultaneous appearance of oxidation and reduction peaks in the CV profiles indicates that these multiple π-assemblies exhibit both electron donor and acceptor capabilities.

**Figure 13:**
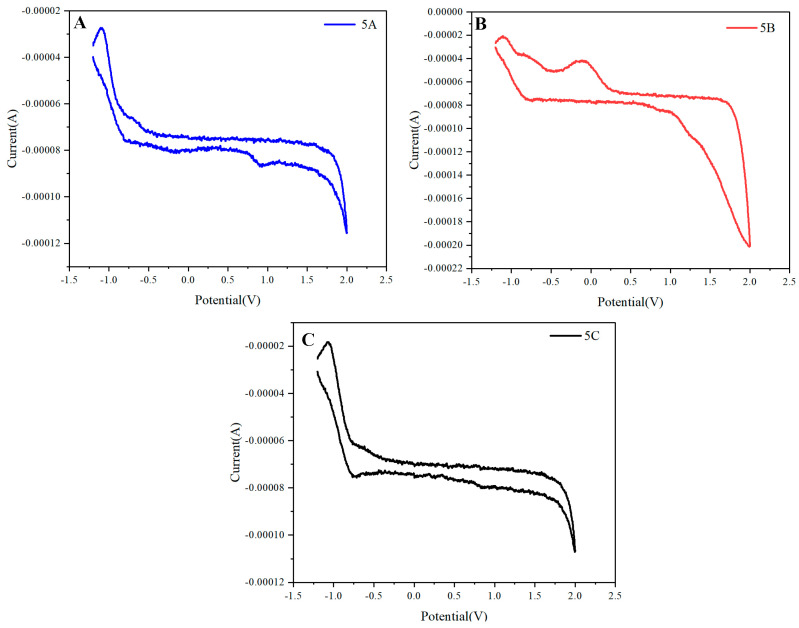
Electrochemical performance of (**A**) **5A**, (**B**) **5B**, and (**C**) **5C** in CH_2_Cl_2_ containing 0.1 M Bu_4_NPF_6_ as an electrolyte at 298 K; the scan rate is 100 mV s^−1^.

The cyclic voltammetry (CV) curves for the naphthalene-based multilayered 3D polymers exhibit two distinct redox peaks, indicating the presence of reversible oxidation and reduction processes. The observation of these peaks suggests that the multiple π-assemblies possess the capability for both electron donation and acceptance. Specifically, the oxidation peak for polymer **5A** occurs at approximately 1.70 V, while the oxidation and reduction maxima for polymer 2 are situated at around 1.5 V and 1.25 V, respectively (Figure 13A). Notably, polymer **5A** demonstrates an oxidation peak at approximately 2.0 V, which indicates a higher energy requirement for electron removal. This observation suggests that polymer **5A** has stronger electron-donating properties or more stable radical cations compared to the other polymers. The reduction peak observed at around 0.9 V further reflects polymer **5A**’s ability to accept electrons, highlighting its electrochemical stability. In contrast, polymers 5B and 5C exhibit their oxidation and reduction peaks at approximately 1.70 V and 1.0 V, respectively. These lower energy thresholds for both oxidation and reduction processes suggest enhanced charge transport properties or lower ionization energy for these polymers compared to polymer **5A**. It is important to clarify the nature of these processes: while the curves may exhibit multiple peaks, they are indeed reversible within the measured potential range, consistent with the cyclic voltammetry analysis. Additionally, the reference electrode used for these measurements is Ag/AgNO_3_ (non-aqueous), providing a reliable reference point for oxidation and reduction potentials. Thus, the observed oxidation peaks at higher potentials are not artifacts of solvent effects but rather reflective of the intrinsic electrochemical properties of the polymers.

## 4. Conclusions

In conclusion, this study successfully synthesizes novel multilayer 3D chiral polymers using 2,2′-(2,7-Naphthalenediyl)bis[4,4,5,5-tetramethyl-1,3,2-dioxaborolane] and 1,8-dibromonaphthalene as key precursors. Comprehensive characterization through nuclear magnetic resonance (NMR), gel permeation chromatography (GPC), photoluminescence, ultraviolet (UV) spectroscopy, scanning electron microscopy (SEM), polarimetry, dynamic light scattering (DLS), thermogravimetric analysis (TGA), and cyclic voltammetry (CV) has provided significant insights into their structural and functional properties. The polymers demonstrate remarkable aggregation-induced emission (AIE) and aggregation-induced polarization (AIP), underscoring their potential for advanced applications in optoelectronics and sensing technologies. Future work should explore the mechanisms behind AIE and AIP and the integration of these polymers into multicomponent reactions [34,35,36,37] and new functional systems [38] advancing the field of chiral polymer research.

## Figures and Tables

**Table 1:** Results of synthetic multilayer 3D chiral polymer.

Poly-Prod	Yield [%] ^a^	M_w_ ^b^	M_n_ ^b^	PDI ^c^	${\left[\alpha \right]}_{D}^{20}$ ^d^	Theor. Layers ^e^
**5A**	68	115,453	33,934	3.402	+24 (c = 0.1)	89
**5B**	61	116,561	33,209	3.510	+11 (c = 0.1)	76
**5C**	55	116,849	33,272	3.512	+3 (c = 0.1)	66

^a^ Isolated yield based on a co-monomer for each case (co-monomers’ ratio = 1:1). ^b^ Determined by GPC with a polystyrene standard (2 mg/mL). ^c^ PDI = *M*_w_/*M*_n_. ^d^ g/100 mL_._ ^e^ Calculated based on M_n_ of GPC determination.

## Data Availability

The data are contained within this article and its Supplementary Materials.

## Supplementary Materials

The following supporting information can be downloaded at https://www.mdpi.com/article/10.3390/molecules30071567/s1, Figure S1. NMR spectra of polymer **5A**. Figure S2. NMR spectra of polymer **5B**. Figure S3. NMR spectra of polymer **5C**. Figure S4. GPC of polymer **5A**. Figure S5. GPC of polymer **5B**. Figure S6. GPC of polymer **5C**.
