# Supplementary material for: Synthesis and Characterization of Multilayer 3D Chiral Polymers with Enhanced Optical Properties
Source: Molecules. 2025 Mar 31;30(7):1567. doi: 10.3390/molecules30071567 (PMC11990258; doi:10.3390/molecules30071567)
Supplement: Supplementary file 1 [file molecules-30-01567-s001.zip › molecules-3512277-supplementary.pdf]

Synthesis and Characterization of Multilayer 3D Chiral Polymers with Enhanced Optical Properties

Sai Zhang,[1,*] Xiaobei Jin,[2] Daixiang Chen,[1] Qingzheng Xu,[3] Tao Wang,[1] Xiuyuan Qin,[4] Jialing Mao,[5] Yue Zhang,[5] Shenghu Yan,[1] and Guigen Li [6,*]

[1]School of Pharmacy, Changzhou University, Changzhou 213164, China;

[2]Changzhou Tronly Advanced Electronic Materials Co. Ltd.

[3]School of Chemistry and Chemical Engineering, Nanjing University, Nanjing 210093, China

[4]School of Life and Science, Nanjing Normal University, Nanjing 210046, China

[5]School of Environmental Science and Engineering, Changzhou University, Changzhou 213164, China

[6]Department of Chemistry and Biochemistry, Texas Tech University, Lubbock, TX 79409-1061, USA

*Correspondence: zhangsai@cczu.edu.cn; guigen.li@ttu.edu

1. NMR spectra and GPC spectra of polymer **5A**,**5B** and **5C**

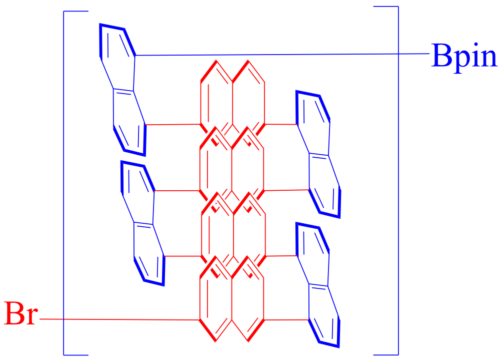

5A

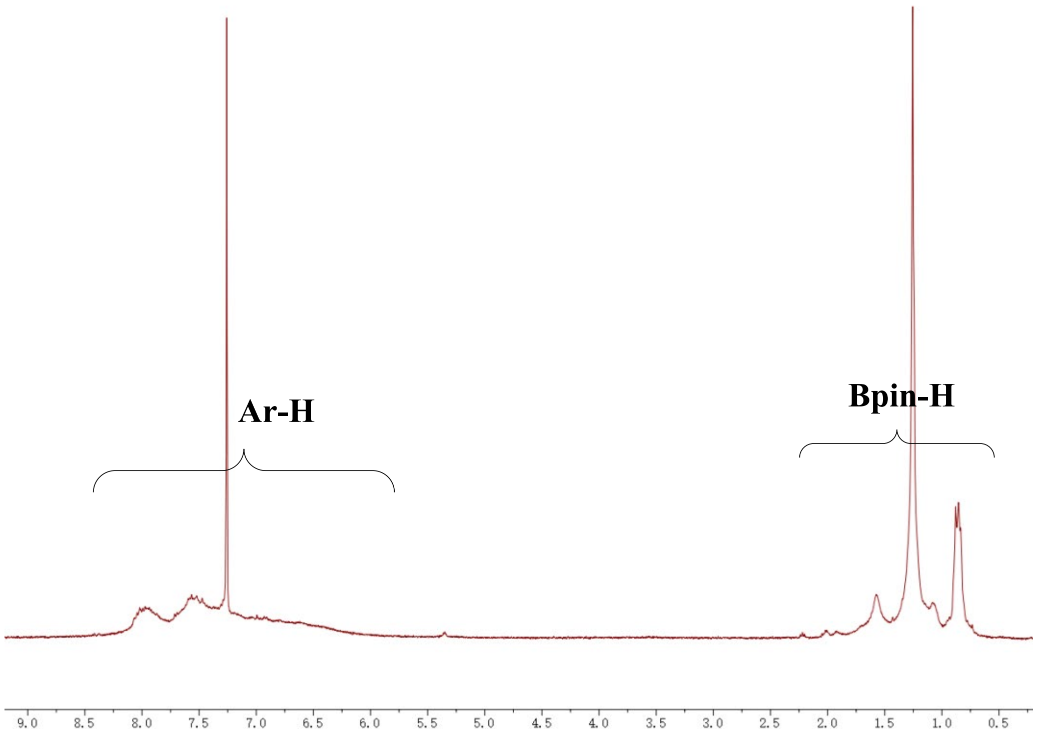

Figure S1. NMR spectra of polymer **5A**

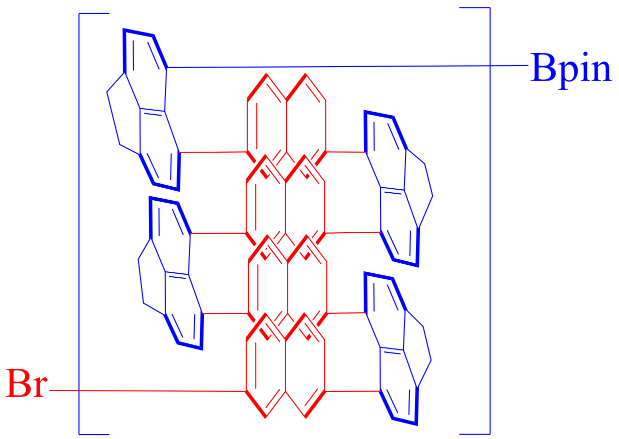

5B

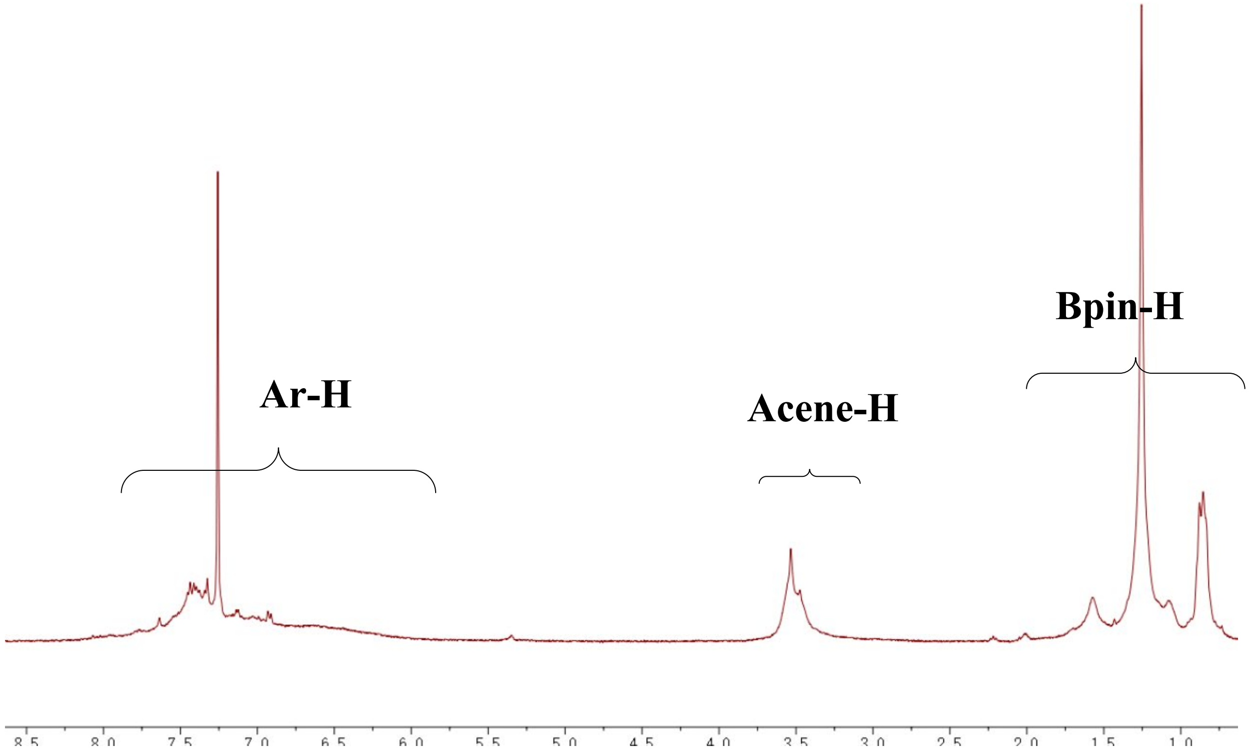

Figure S2. NMR spectra of polymer **5B**

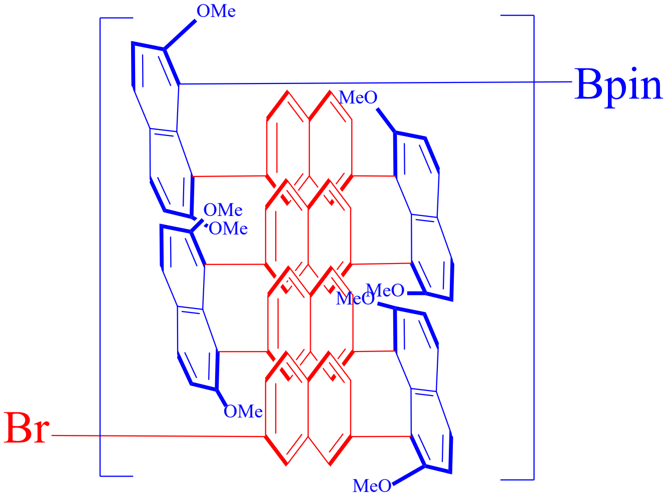

5C

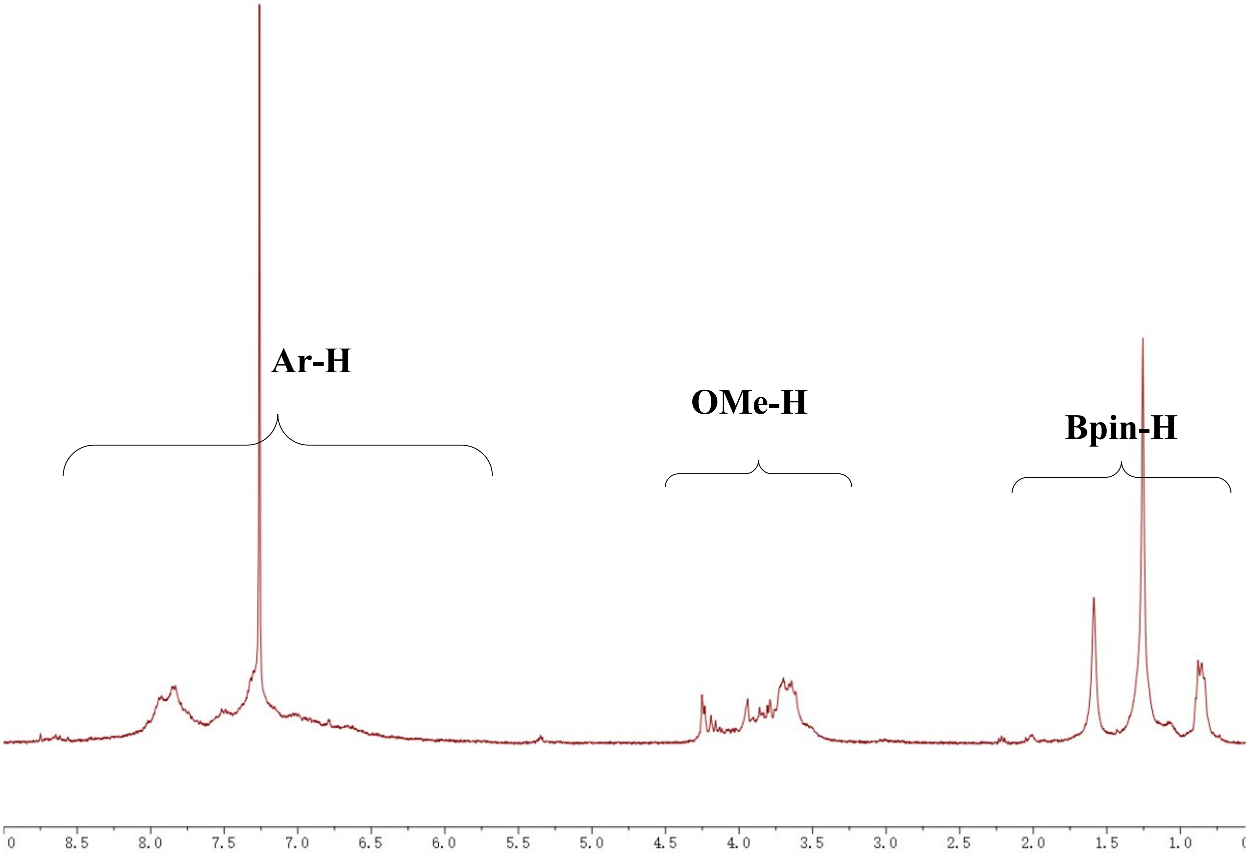

Figure S3. NMR spectra of polymer **5C**

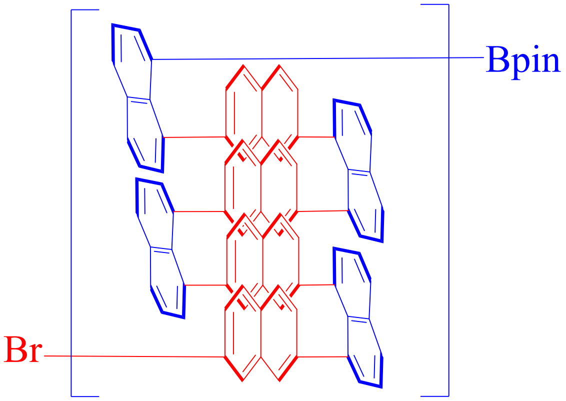

5A

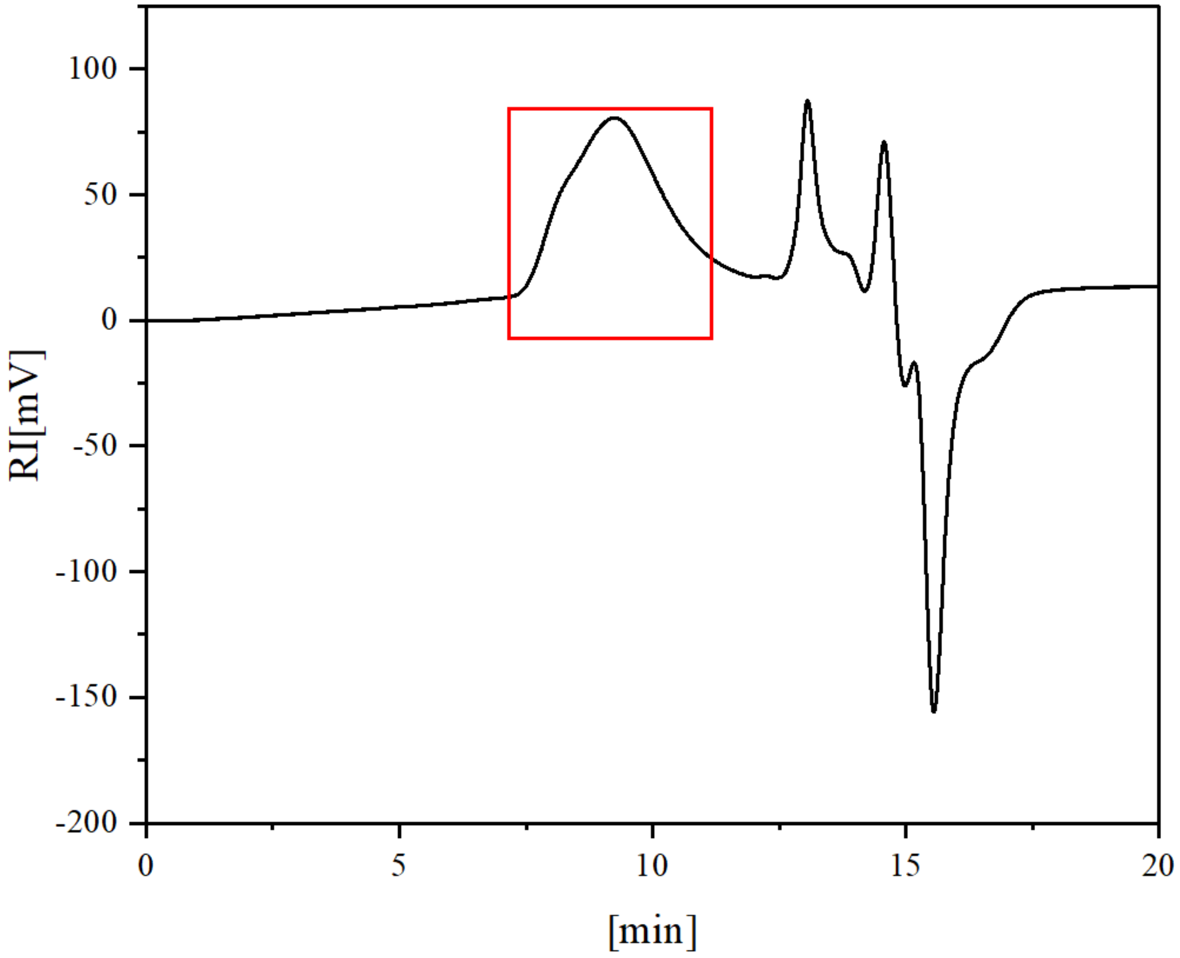

Figure S4. GPC of polymer **5A**

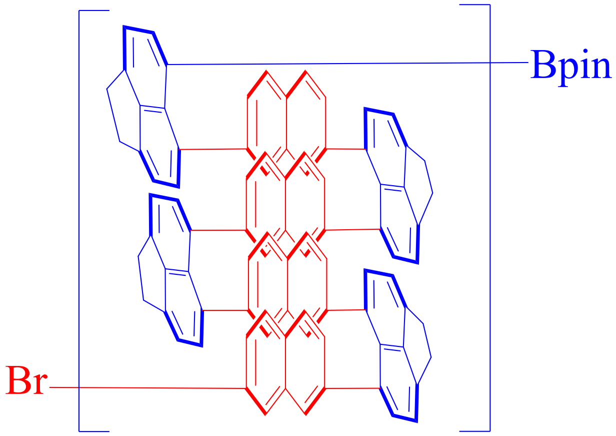

5B

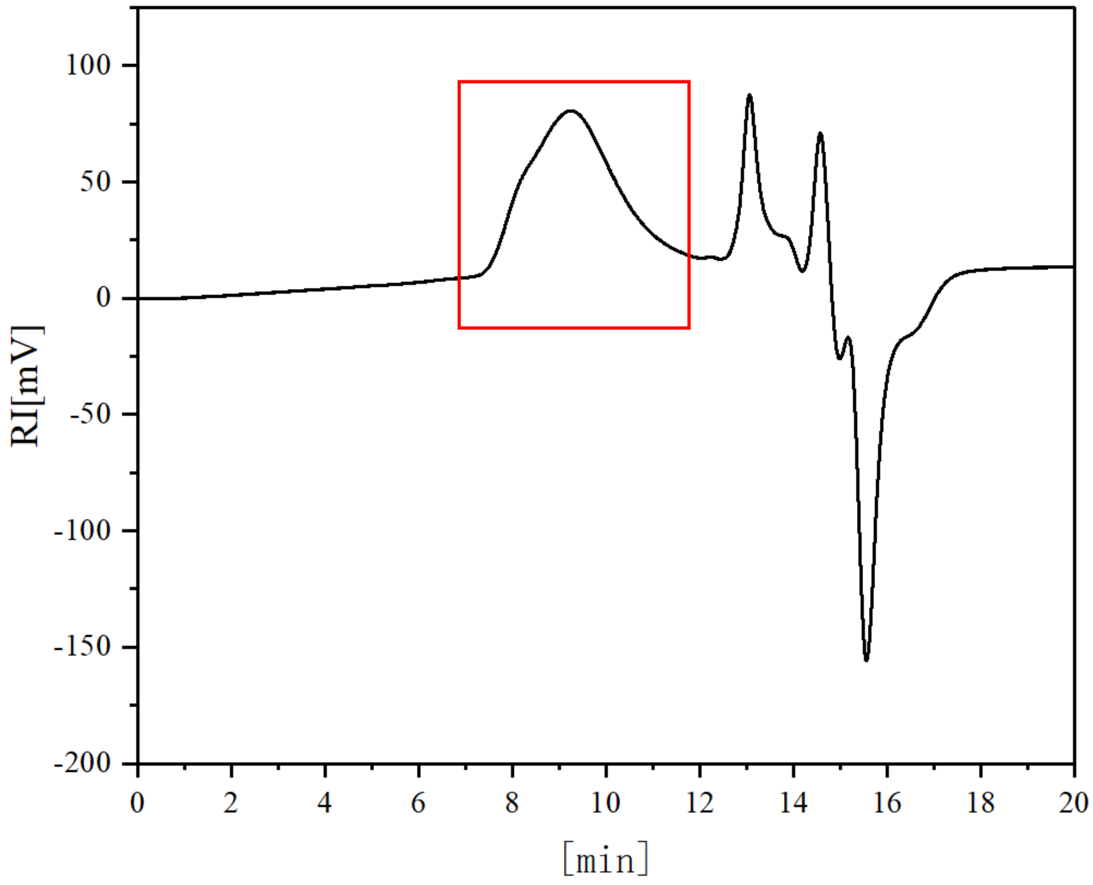

Figure S5. GPC of polymer **5B**

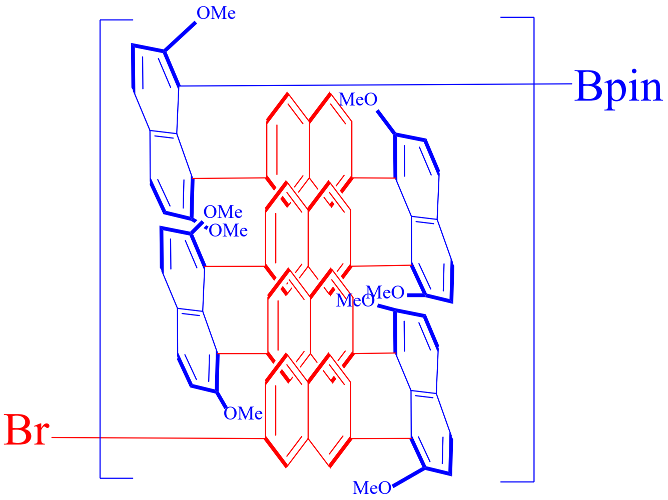

5C

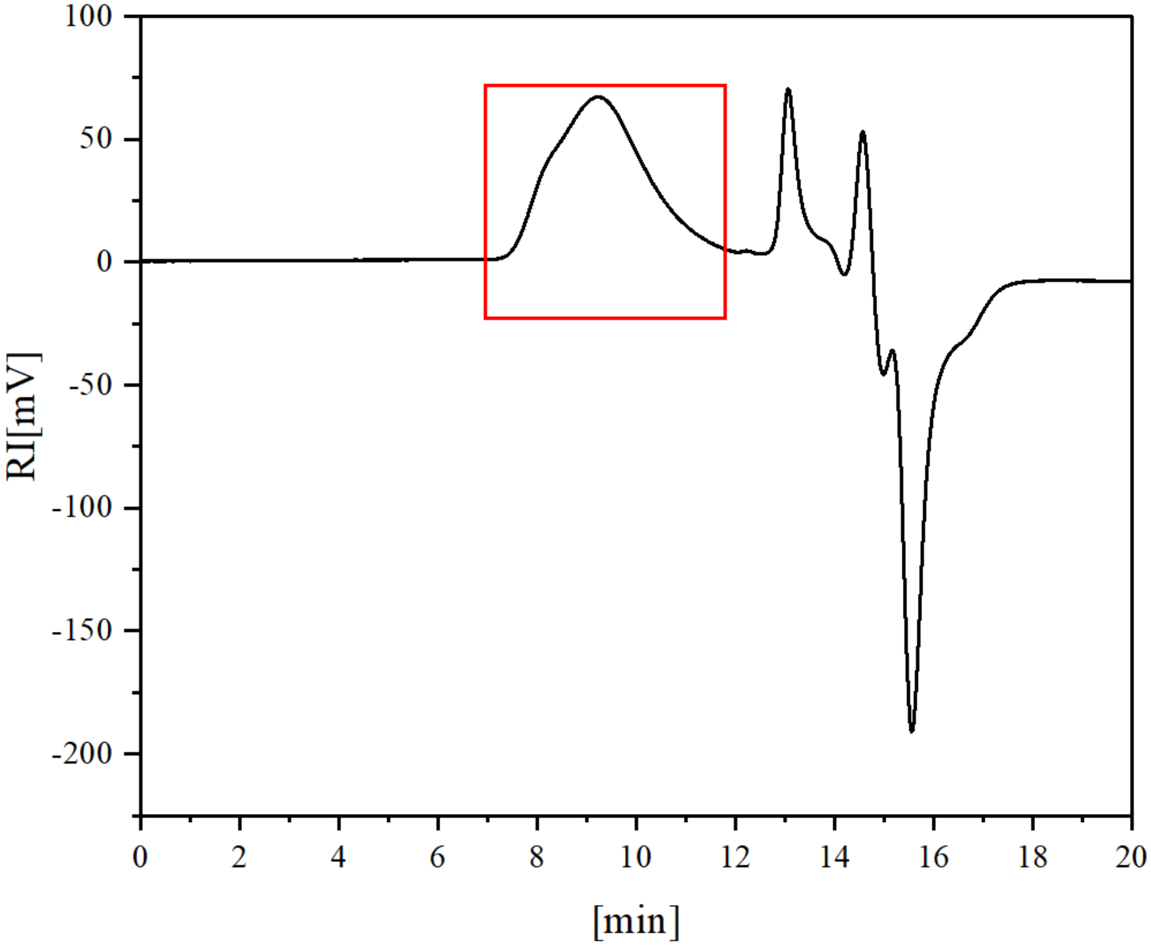

Figure S5. GPC of polymer **5B**
